# Estimating probabilities of peptide database identifications to LC-FTICR-MS observations

**DOI:** 10.1186/1477-5956-4-1

**Published:** 2006-02-24

**Authors:** Kevin K Anderson, Matthew E Monroe, Don S Daly

**Affiliations:** 1 Computer Science and Mathematics Division, Pacific Northwest National Laboratory, Battelle Boulevard, Richland WA, USA; 2 Biological Sciences Division, Pacific Northwest National Laboratory, Battelle Boulevard, Richland WA, USA

## Abstract

**Background:**

The field of proteomics involves the characterization of the peptides and proteins expressed in a cell under specific conditions. Proteomics has made rapid advances in recent years following the sequencing of the genomes of an increasing number of organisms. A prominent technology for high throughput proteomics analysis is the use of liquid chromatography coupled to Fourier transform ion cyclotron resonance mass spectrometry (LC-FTICR-MS). Meaningful biological conclusions can best be made when the peptide identities returned by this technique are accompanied by measures of accuracy and confidence.

**Methods:**

After a tryptically digested protein mixture is analyzed by LC-FTICR-MS, the observed masses and normalized elution times of the detected features are statistically matched to the theoretical masses and elution times of known peptides listed in a large database. The probability of matching is estimated for each peptide in the reference database using statistical classification methods assuming bivariate Gaussian probability distributions on the uncertainties in the masses and the normalized elution times.

**Results:**

A database of 69,220 features from 32 LC-FTICR-MS analyses of a tryptically digested bovine serum albumin (BSA) sample was matched to a database populated with 97% false positive peptides. The percentage of high confidence identifications was found to be consistent with other database search procedures. BSA database peptides were identified with high confidence on average in 14.1 of the 32 analyses. False positives were identified on average in just 2.7 analyses.

**Conclusion:**

Using a priori probabilities that contrast peptides from expected and unexpected proteins was shown to perform better in identifying target peptides than using equally likely a priori probabilities. This is because a large percentage of the target peptides were similar to unexpected peptides which were included to be false positives. The use of triplicate analyses with a "2 out of 3" reporting rule was shown to have excellent rejection of false positives.

## Background

**Figure 1:**
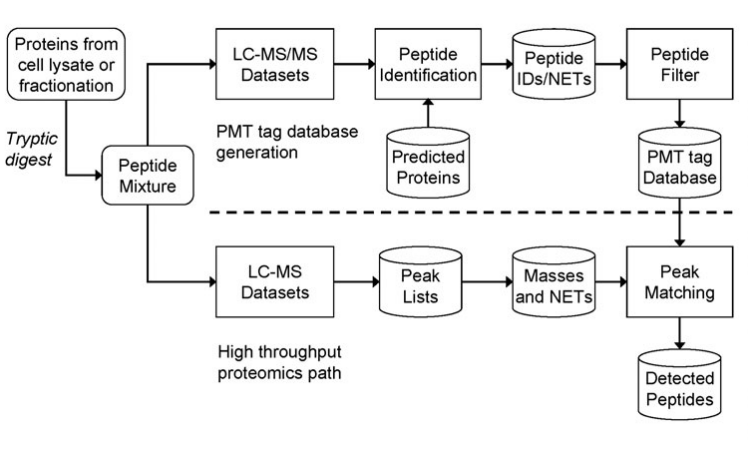
PNNL Proteomics Analysis Pipeline.

The high-throughput determination of the identities and abundances of peptides and proteins in a biological sample is important to systems biology research. Although many different proteomics methodologies are in use today, Pacific Northwest National Laboratory (PNNL) has implemented a high-throughput process based on multiple mass spectrometry technologies, including liquid chromatography coupled to Fourier transform ion cyclotron resonance mass spectrometry (LC-FTICR-MS) [1-4]. This high-throughput process is diagramed in Figure 1.

The PNNL proteomic analysis process consists of two major steps: 1) generation of a potential mass and time (PMT) tag database, created from experimental data, containing all of the peptide detections for the organism being studied and 2) high-throughput LC-FTICR-MS experiments to identify the peptides and proteins expressed under various biological conditions, using the PMT tag database for identification. The database is generated through a series of tandem mass spectrometry (LC-MS/MS) experiments, using the resultant data to build a comprehensive set of peptides likely to be expressed by the organism under various growth and stress conditions. Within the PMT tag database, each peptide has an exact mass (calculated from its amino acid sequence), an observed normalized elution time (NET), and various confidence indicators associated with it, all resulting from the analysis of the MS/MS fragmentation spectra.

The high mass accuracy of LC-FTICR-MS allows peptide identifications to be made by searching the PMT tag database with the masses observed in a deisotoped mass spectrum. The search scope can be narrowed further using the normalized elution time of the given spectrum [1]. Automated database searches are required for high-throughput proteomic sample analyses. For LC-MS/MS analyses, search software that makes use of scores to reflect the degree of fit between the MS data and the peptides in the database has been developed (for example [5]). In the case of LC-FTICR-MS analyses, probabilities can be estimated to the matches, providing a direct measure of the confidence of peptide identifications. We present a statistical methodology and the underlying assumptions used to estimate probabilities to the matches of LC-FTICR-MS features to known peptides in a database. Norbeck et. al. have used this method to explore the specificity of theoretical tryptic digests from complex proteomes [6]. Our search methodology compares the observed masses and the normalized elution times of the detected features to the theoretical masses and normalized elution times of the known peptides in the database. We demonstrate our methodology using 32 LC-FTICR-MS analyses of a tryptically digested bovine serum albumin (BSA) sample and a database consisting of 72,962 peptides.

Our work was first reported at METMBS'04 [7], where we focused on the statistical derivation and the equally likely prior probability case illustrated with a simple example. Since then, we have expanded our focus: refining our motivation, improving the statistical algorithms, and applying these in a more complex example using the prior probabilities for rejection of false positives. We describe our most recent work in this paper.

## Methods

The problem of estimating the probability that a peptide in an appropriate PMT tag database matches the measurements of a feature from an LC-FTICR-MS analysis is a statistical classification problem. That is, the measurements come from one of a number of known statistical populations, but from which did they arise? We assume that the PMT tag database establishes the entirety of possible populations. Although a statistical classification problem is formally a Bayesian statistical decision problem involving losses, costs of misclassification, and *a priori *(prior) probabilities, we will concern ourselves with calculating the conditional probabilities of coming from each of the possible populations, given the measurement [8]. These conditional probabilities will reflect our confidence that the peptides are in the sample analyzed by LC-FTICR-MS. A peptide is considered to be identified if the conditional probability is large enough; here, 0.95 is taken to be high confidence.

To begin our development of the conditional probabilities, suppose **M**_*i *_is the vector of measured mass and normalized elution time of the *i*-th feature from the LC-FTICR-MS analysis, **M**_*i *_= (*m*_*i*_, *t*_*i*_). Let *μ*_*j *_= (*μ*_*mj*_, *μ*_*tj*_) be the vector of theoretical mass and elution time from the *j*-th record (peptide) of the PMT tag database. The subscripts *m *and *t *denote mass and normalized elution time, respectively.

If we assume that if the *i*-th feature was the same as the *j*-th record, then **M**_*i *_is statistically distributed as a bivariate Gaussian distribution with mean vector *μ*_*j *_and covariance matrix Σ_*j*_. As a first-order approximation, suppose that mass and normalized elution time are independent, so that Σ_*j *_is a diagonal matrix with diagonal elements  and , where these variances reflect the uncertainties in measured LC-FTICR-MS masses and normalized elution times.

A standardized distance of the measurement **M**_*i *_to *μ*_*j *_is



under the assumption of independence between the measurements of mass and normalized elution time. If we knew the prior probability *π*_*j *_that measurements come from the distribution associated with the *j*-th record, then the conditional probability that **M**_*i *_comes from the *j-*th record, given the measurement **M**_*i*_, is



where *N *is the number of peptides in the PMT tag database and the determinant |Σ_*j*_| = . This paper will compare the results using prior probabilities with the results assuming equally likely prior probabilities (i.e., *π*_*j *_= *π*_*k *_for all *j *and *k*), which yields conditional probabilities



The prior probabilities in Equation (2) are meant to reflect the analysts' expectation of observing the particular peptides among the sample's observed LC-FTICR-MS features. The prior probabilities can account for the likelihood that proteins are present in the sample and, at a more detailed level, the detectabilities of the peptides of each protein (which vary because of mass/charge effects).

If we drop the assumption that the PMT tag database establishes the entirety of possible peptides in the analyzed sample, we can admit the possibility that a LC-FTICR-MS feature is something else when the *d*_*ij *_values are large for all records in the PMT tag database. Equation (3) will always assign probabilities, but if *d*_*ij *_is greater than 10.6 (approximately the 99.5th percentile of the chi-squared distribution with 2 degrees of freedom), we will consider the *i*-th feature as unmatched.

## Results

We matched 32 LC-FTICR-MS analyses of a tryptically digested bovine serum albumin (BSA) sample to a PMT tag database consisting of 72,962 PMT tags (peptides). The PMT tag database was constructed by analyzing a tryptically digested sample of a mixture of standard proteins and peptides 318 times over a ten-month period using LC-MS/MS. This mixture, containing BSA, 11 other proteins, and 23 peptides, was recently proposed as a quality assessment standard for use in proteomics studies [9]. In order to populate the database with false positive results, the LC-MS/MS data was searched using an organism database file combining both known protein sequences and sequences from *S. Oneidensis *(Shewanella). The resulting PMT tag list contains three distinct types of peptides: 1) bovine peptides (including BSA), 2) peptides from known, standard proteins, and 3) false positive Shewanella peptides (97.1% of the PMT tag list). Thus, the ability of the methodology to reject spurious matches can be tested.

The software package SEQUEST [10] was used to identify candidate peptides for the fragmentation spectra. The organism database file provided to SEQUEST contained the sequences of BSA, as well as the other 11 proteins and 23 peptides in the protein standard mixture, 4 variants of human keratin (a common protein contaminant from sample preparation), and 14 other standard proteins chosen because they are commonly used in other protein standard mixtures. In addition, the organism database file contained the sequences for the 4897 Shewanella proteins obtained from the sequence repository at TIGR (March 21, 2000). This gave the following distribution of amino acid residues: 0.27% from bovine peptides, 0.54% from standard proteins and known contaminants, and 99.2% from Shewanella and other unexpected proteins.

The SEQUEST search was performed with enzyme rules turned off, thus allowing partially and non-tryptic peptides to be identified, although nontryptic peptides were excluded from the PMT tag database. The PMT tag database was populated with the sequences having cross correlation (XCorr) values > 1.9 and delta correlation (DelCn) values < 0.10. Due to the inherent false positive rate in the sequences determined by SEQUEST, the peptides that were observed in only one MS/MS fragmentation spectrum were required to have higher XCorr values: > 1.9 for 1+ charge species, > 2.2 for 2+ species, and > 3.5 for 3+ species (note that 164,461 MS/MS spectra contained at least one valid peptide vs. 715,724 total MS/MS spectra). The peptide monoisotopic masses *μ*_*mj *_in the PMT tag database were computed theoretically. The normalized elution times (NETs) *μ*_*tj *_were determined empirically from the observed LC-MS/MS elution times (using discriminant scores as weights [11]).

The peptides in the PMT tag database were examined for identification ambiguities: peptides found in multiple proteins or with similar PMT tags. Only three peptide sequences were found to be common to two different protein sources each (one rabbit and two horse peptides were also Shewanella peptides). One-third of the BSA peptides (60 out of 179) were close in the standardized distance metric to some other peptide in the PMT tag database; 57 were close to a Shewanella peptide, one was close to a peptide from another bovine protein, and one pair of BSA peptides was close to each other. High-confidence identification of these 60 BSA peptides is unlikely.

The LC-FTICR-MS analyses were processed using the PRISM Data Analysis system, a series of software tools developed in-house. The first step involved deisotoping the MS data, giving the monoisotopic mass, charge, and intensity of the major peaks in each mass spectrum. Following this, the data were examined in a two-dimensional fashion to determine the sets of mass spectral peaks that make up each peptide's charge-state profile. Each feature (set of peaks) represents a single component eluting from the LC column and detected by the mass spectrometer. The feature's inherent properties are median mass, central normalized elution time (NET), and an abundance estimate determined using the area of the MS peaks belonging to the most abundant charge state in the feature. Features with NET values ≥ 0.9 were omitted from analysis as such features are most likely mass calibration compounds that are infused at the completion of each analysis.

The identity of each of the 69,220 features in the 32 LC-FTICR-MS analyses of the BSA sample was determined by comparing the mass and NET of each feature with the mass and NETs of the 72,962 PMT tags in the database. The standardized distances of Equation (1) were computed assuming 3 parts per million uncertainty on mass, that is, *σ*_*mj *_= 0.000003*μ*_*mj*_, and setting *σ*_*tj *_equal to 0.025 added in quadrature to the standard error of the empirically determined *μ*_*tj*_. Thus, *σ*_*tj *_reflects the uncertainty of *μ*_*tj *_in the PMT tag database and the LC-FTICR-MS NET measurement error. Three cases were considered in the calculation of conditional probabilities, the first being our proposed procedure and two others for comparison.

**Case 1: **conditional probabilities were calculated using Equation (2) with prior probabilities set according to whether or not the PMT was from an expected protein source with a ratio of 50:1, respectively. The expected sources for the 32 analyses of the BSA sample were BSA, other bovine proteins, trypsin, markers, and common human contaminants (keratin variants).

**Case 2: **conditional probabilities were calculated using Equation (3) with equally likely prior probabilities.

**Case 3: **like Case 1, but with the Shewanella peptides removed from the PMT tag database (effectively setting their prior probabilities to zero and not calculating their Equation (1) *d*_*ij *_value).

At first glance, it would seem that Case 3 would be the most relevant when analyzing BSA samples: remove from the PMT tag database those peptides from completely unexpected sources. However, nearly the same "downweighting" can be accomplished using the prior probabilities of Case 1 without completely taking away the opportunity to find those peptides if they are actually present. There are quality control reasons for wanting large PMT tag databases (to detect cross-contamination of samples, for example). Also, if analyzing unknown samples, extremely large PMT tag databases are required and equally likely prior probabilities are used to represent the lack of information regarding the sample.

### Case 1 results

A large percentage of the features from the 32 LC-FTICR-MS analyses of the BSA sample went unmatched in the Case 1 analysis. Of the 69,220 features, 12,287 (17.8%) passed the d_*ij *_< 10.6 threshold for matching. These 12,287 features were matched with one or more of 9290 of the 72,962 PMT tags with conditional probability of matching greater than 1/1000. 7083 features (10.2%) were identified with high confidence (that is, with a conditional probability greater than 0.95) and are summarized in Table 1, leaving 5204 features as ambiguously matched. Of the 179 BSA PMT tags, 47 were identified in at least 1 of the 32 analyses. While a large proportion of the feature identifications were with the false positive Shewanella peptides, 79.7%, those results were generally not reproducible as the peptides were identified on average in just 2.7 of the 32 analyses. Contrast that with the BSA identifications that were identified on average in 14.1 of the 32 analyses. PNNL typically runs samples in triplicate and applies a "2 out of 3" rule for reporting peptide identifications. Based on this reporting rule, 13 false positive Shewanella peptides would be expected to be reported (that is, with probabilities ≥ 0.87 of being identified in at least 2 out of 3 analyses), contrasted with 16 expected BSA peptides. Besides these peptides, of all the other peptides in the PMT tag database, only 2 peptides from common human contaminants and 1 marker peptide would be expected to be reported. While the expected reported number of false positives is large compared to the expected reported number of the target BSA peptides, the false positive rejection is excellent considering 97.1% of the PMT tags were false positives and 0.2% were the targets.

**Table 1:** High confidence peptide identifications across 32 analyses using 50:1 relative prior probabilities.

Protein source (# of PMT tags)	Number of PMT tags observed	Average number of identifications	feature identifications
BSA (179)	47	14.1	10.4%
Bovine (607)	72	2.6	3.0%
Human (385)	37	4.8	2.8%
Marker (18)	12	6.9	1.3%
Trypsin (24)	6	6.8	0.6%
Shewanella (70,832)	1873	2.7	79.7%
Other (917)	54	2.6	2.2%

### Case 1 results compared to Cases 2 and 3

Summaries of the high confidence BSA peptide identifications from the Case 2 and Case 3 analyses are presented in Tables 2 and 3. The Case 2 analysis has the same unmatched features as Case 1 (82.2%) because the *d*_*ij *_values of Equation 1 do not depend on the prior probabilities used in the subsequent conditional probability calculations. The Case 2 analysis identifies 27.7% fewer BSA peptides across the 32 analyses than the Case 1 analysis because of the large number of BSA peptides that are ambiguous with other peptides (mostly Shewanella peptides as mentioned above). The Case 3 analysis leaves a larger percentage, 97.1%, of the features unmatched because of the significantly smaller PMT tag database. However, nearly all (99.2%) of the matches are high confidence and 14.9% more BSA peptides are identified across the 32 analyses because the ambiguous Shewanella peptides are removed from consideration. When BSA feature matches with conditional probabilities ≥ 0.6 are considered, there is 98.5% agreement between the Case 1 and Case 3 analyses (685 features out of 696 and 695 features, respectively).

**Table 2:** High confidence peptide identifications across 32 analyses using equally likely prior probabilities.

Protein source (# of PMT tags)	Number of PMT tags observed	Average number of identifications	feature identifications
BSA (179)	34	13.8	7.6%
Bovine (607)	54	2.8	2.4%
Human (385)	28	5.3	2.4%
Marker (18)	12	6.7	1.3%
Trypsin (24)	2	3.5	0.1%
Shewanella (70,832)	1892	2.7	83.9%
Other (917)	54	2.6	2.2%

**Table 3:** High confidence peptide identifications across 32 analyses using 50:1 relative prior probabilities without the Shewanella peptides in the PMT tag database

Protein source (# of PMT tags)	Number of PMT tags observed	Average number of identifications	feature identifications
BSA (179)	54	12.9	43.1%
Bovine (607)	94	3.0	17.7%
Human (385)	41	5.0	12.6%
Marker (18)	12	6.9	5.1%
Trypsin (24)	6	6.8	2.5%
Other (917)	105	2.9	19.0%

The average number of analyses in which PMT tags were identified was similar across analysis cases. Considering the "2 out of 3" reporting rule, for the Case 2 analysis, 10 BSA, 13 Shewanella, 2 human keratin, and 1 marker peptides would be expected to be reported. For the Case 3 analysis, 17 BSA, 1 bovine, 1 *E. coli*, 2 human keratin, and 1 marker peptide would be expected to be reported.

## Conclusion

Validation of the estimation of conditional probabilities is difficult because of the lack of ground truth: protein samples deemed to be pure often contain 10% impurities/contaminants while peptide digestion can be incomplete resulting in missed cleavages.

The use of prior probabilities set to contrast peptides from expected/unexpected proteins was shown to perform better in identifying target peptides than using equally likely prior probabilities. This is because a large percentage of the target peptides (BSA) were similar in PMT tag space to unexpected peptides (Shewanella) which were chosen to be false positives in the PMT tag database. Equally likely prior probabilities would perform well when there is little or no overlap. When using the contrasting prior probabilities, a large percentage, 81.9%, of our high confidence feature identifications were of peptides from unexpected sources (false positives). Using equally likely prior probabilities was only a bit worse with 86.1% unexpected identifications. However, in both cases, these unexpected identifications were not reproducible, appearing in less than 3 out of 32 analyses on average.

The completeness of the PMT tag database is also an issue, as 82.3% of the features were unmatched, that is, were not close to any of the PMT tags in the monoisotopic mass/normalized elution time space. However, this matching rate is similar to that of other PMT tag database search procedures.

Examination of the proteins to which the matched peptides belong could be used to improve the computed conditional probability. For example, the probability for those peptides derived from proteins with multiple peptide matches should be higher than that for those peptides derived from proteins with single or few peptide matches. While we took into account the uncertainty of the empirically determined elution times, one might also incorporate into the matching procedure the SEQUEST XCorr or any other quality of identification score for the peptides in the PMT tag database. Finally, comparison of the observed NET with the predicted NET for the peptide [12] could lead to a further improvement in the utility of the conditional probabilities.

## Authors' contributions

DSD, KKA and MEM detailed the physical problem of assigning peptide labels to LC-FTICR observations. KKA and DSD translated the physical description into a statistical problem, resolved all statistical issues and translated statistical results into physical statements. KKA suggested the particular statistical classification approach, derived the appropriate algorithms, developed prototype code, conducted the data analyses, and produced the featured statistical results. MEM extracted, filtered, packaged and then delivered the proteomics data sets. DSD, KKA and MEM determined an appropriate biological interpretation. DSD conceived and managed the study, assembled the initial draft, and served as the primary editor. KKA was the principal author of the manuscript. All authors submitted comments on drafts, then read and approved the final manuscript.

## Declaration of competing interests

The author(s) declare that they have no competing interests.
